# Changes in Medial Prefrontal Cortex Mediate Effects of Heart Rate Variability Biofeedback on Positive Emotional Memory Biases

**DOI:** 10.1007/s10484-023-09579-1

**Published:** 2023-01-20

**Authors:** Christine Cho, Hyun Joo Yoo, Jungwon Min, Kaoru Nashiro, Julian F. Thayer, Paul M. Lehrer, Mara Mather

**Affiliations:** 1 University of Southern California, 3715 McClintock Ave., Los Angeles, CA 90089 USA; 2 University of California, Irvine, USA; 3 Rutgers Robert Wood Johnson Medical School, New Brunswick, USA

**Keywords:** Heart rate variability, Emotion regulation, Emotional memory, Memory bias, Amygdala, Medial prefrontal cortex

## Abstract

Previous research suggests that implicit automatic emotion regulation relies on the medial prefrontal cortex (mPFC). However, most of the human studies supporting this hypothesis have been correlational in nature. In the current study, we examine how changes in mPFC-left amygdala functional connectivity relate to emotional memory biases. In a randomized clinical trial examining the effects of heart rate variability (HRV) biofeedback on brain mechanisms of emotion regulation, we randomly assigned participants to increase or decrease heart rate oscillations while receiving biofeedback. After several weeks of daily biofeedback sessions, younger and older participants completed an emotional picture memory task involving encoding, recall, and recognition phases as an additional measure in this clinical trial. Participants assigned to increase HRV (Osc+) (*n* = 84) showed a relatively higher rate of false alarms for positive than negative images than participants assigned to decrease HRV (Osc−) (*n* = 81). Osc+ participants also recalled relatively more positive compared with negative items than Osc− participants, but this difference was not significant. However, a summary bias score reflecting positive emotional memory bias across recall and recognition was significantly higher in the Osc+ than Osc− condition. As previously reported, the Osc+ manipulation increased left amygdala-mPFC resting-state functional connectivity significantly more than the Osc− manipulation. This increased functional connectivity significantly mediated the effects of the Osc+ condition on emotional bias. These findings suggest that, by increasing mPFC coordination of emotion-related circuits, daily practice increasing heart rate oscillations can increase implicit emotion regulation.

**Supplementary Information:**

The online version contains supplementary material available at 10.1007/s10484-023-09579-1.

## Introduction

Previous studies have found that the ventromedial prefrontal cortex (vmPFC) plays a key role in regulating both heart rate variability (HRV) and emotions (Thayer et al., 2012). This region encompasses both the medial and lateral sectors of the orbitofrontal cortex and extends through the anterior cingulate (Bechara, 2004). Individual differences in vagally mediated HRV are more associated with both function and structure in ventromedial PFC than with most other brain regions (Koenig et al., 2021; Sakaki et al., 2016; Thayer et al., 2012; Yoo et al., 2018). Based on the famous case of Phineas Gage and other patients with vmPFC lesions, the vmPFC has long been identified as playing a role in emotion regulation (Bechara, 2004; Beer et al., 2007). Additionally, modern theorizing posits that vmPFC is critical for automatic emotion regulation processes (Braunstein et al., 2017). In contrast, lateral PFC and dorsomedial PFC regions are involved in controlled emotion regulation processes such as those elicited in an instructed emotion regulation task (Braunstein et al., 2017). Another interesting aspect of the vmPFC is that it distinguishes between positive and negative affect more than other emotion-related brain regions. In a meta-analysis of nearly 400 studies, it was the only brain region that consistently showed differential activity between positive and negative affect conditions (Lindquist et al., 2016).

One fascinating question is whether some brain circuits regulate both heart rate and emotion. Consistent with this possibility, higher heart rate variability (HRV) is associated with more effective emotion regulation (Pinna & Edwards, 2020). Furthermore, studies using HRV biofeedback suggest a causal link between HRV and emotion regulation. Meta-analyses across studies indicate that daily biofeedback sessions designed to increase the spectral power of heart rate oscillatory activity via slow paced breathing (i.e., HRV biofeedback) decrease stress, anxiety, depression and anger (Goessl et al., 2017; Lehrer et al., 2020; Pizzoli et al., 2021). These intervention studies raise the possibility that daily sessions involving high amplitude in heart rate oscillatory activity increase the strength of physiological feedback loops involving vmPFC, and that the improved vmPFC function in turn leads to more effective automatic regulation of emotions.

**Fig. 1:**
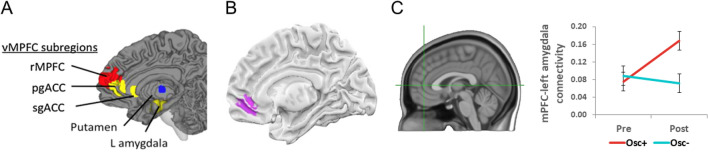
Ventromedial PFC is associated with both HRV and positive affect. Ventromedial PFC is both associated with heart rate variability (HRV) **A** (Thayer et al., 2012), and was the only brain region identified in a meta-analysis of nearly 400 neuroimaging studies as showing more activity during positive than negative affect. **B** (Lindquist et al., 2016). In our recent randomized clinical trial, we found that an mPFC ROI **C** (Nashiro et al., 2022) with coordinates based on (**A**) increased in its functional connectivity with the left amygdala at rest across a 5-week HRV-biofeedback intervention condition compared with an active control condition

In a recent randomized 7-week clinical trial (Clinicaltrials.gov NCT03458910 “Heart Rate Variability and Emotion Regulation” or HRV-ER), we tested the hypothesis that 5 weeks of HRV biofeedback would affect resting amygdala-mPFC functional connectivity (Nashiro et al., 2022), reflecting increasing mPFC involvement in an important emotion regulation circuit. In the target intervention condition, we used resonance paced breathing and HRV biofeedback to increase heart rate oscillations during 20–40 min of daily sessions (Osc+ condition). Resonance paced breathing refers to slow paced breathing at around 10 s per breath, which maximizes oscillations in heart rate due to resonance with baroreflex oscillatory influences on heart rate (Vaschillo et al., 2006). In an active control condition, participants also practiced HRV biofeedback but were instructed to try to keep their heart rate steady while relaxing (Osc− condition). We told participants in both conditions that meditation can improve emotional health, reducing stress and anxiety, and that we were interested in whether the effects of meditative practices on heart rate contribute to those effects. To the Osc+ participants, we explained that some meditative practices produce large oscillations in heart rate. To the Osc− participants, we explained that some meditative practices produce a low and steady heart rate. Thus, participants in both conditions believed that we expected them to benefit emotionally from the practice. Among the younger adult cohort, both conditions showed significant improvements in self-rated mood and depression, possibly due to demand effects. Right amygdala-mPFC resting-state functional connectivity showed no significant intervention effects. However, left amygdala-mPFC resting-state functional connectivity significantly increased for the Osc+ condition and not for the Osc− condition (Nashiro et al., 2022) (Fig. 1).

Participants completed the biofeedback training in daily sessions over the course of several weeks before completing a recall and recognition memory task for positive, negative and neutral pictures. This memory task was an additional measure in our clinical trial (Clinicaltrials.gov NCT03458910; for this task see “Other Outcome Measures” #5–6). The underlying mechanistic model we were interested in testing was that daily sessions involving high heart rate oscillations (Osc+ condition) can affect mPFC function, which in turn affects emotional biases that influence retrieval without conscious effort to favor positive or negative memories. The relative degree of recall and recognition of positive versus negative memories is influenced by automatic emotional biases and can contribute to emotional well-being. For instance, depressed people show autobiographical memory biases favoring retrieval of negative over positive memories, with automatic intrusive memories of negative experiences characterizing the disorder (Dalgleish & Werner-Seidler, 2014). Emotional memory biases persist to some extent beyond depressive episodes, serving as a potential risk factor for relapses (Everaert et al., 2022). Training to focus on positive thoughts while inhibiting negative memories significantly decreased depression in depressed patients (Farahimanesh et al., 2021). Additionally, attentional and memory biases towards positive and away from negative stimuli are one component of effectively regulating emotion (Samide & Ritchey, 2021; Todd et al., 2012).

Thus, by modulating brain circuits known to be involved in emotion regulation (see Mather & Thayer, 2018 for further discussion), increasing heart rate oscillations during daily training sessions could enhance emotion regulation and emotional well-being and therefore bias memory retrieval to be more positive. In the current paper, we report on how the random assignment to biofeedback condition affected positive emotional memory bias and how this bias related to the changes in resting amygdala-mPFC functional connectivity. Specifically, we examined whether Osc+ participants would show a greater “positive > negative” memory bias than Osc− participants and whether the degree of this emotional bias would relate to the intervention-induced change in resting amygdala-mPFC functional connectivity.

## Method

### Transparency and Openness

We report how we determined our sample size, all data exclusions, all manipulations, and all measures in the study, and we follow Journal Article Reporting Standards (Kazak, 2018). Data, analysis, and research materials for this paper are available at https://osf.io/t5s4z/ (Cho, 2022), and raw MRI data are available at https://openneuro.org/datasets/ds003823. Data were analyzed using IBM SPSS Statistics for Windows, version 28.0 (IBM Corp., Armonk, N.Y., USA). This study’s parent clinical trial design was pre-registered: https://clinicaltrials.gov/ct2/show/NCT03458910, with the recognition and recall measures collected here listed as ‘other outcome measures.’ This study was approved by the Institutional Review Board at the University of Southern California.

### Participants

Informed consent was obtained from all individual participants included in the study. One hundred and ninety-three participants were recruited for the study, younger adults (YA) between the ages of 18 and 35 years (*N* = 121, *M* = 22.68, 50.4% female, 49.6% male) and older adults (OA) between the ages of 55 and 80 years (*N* = 72, *M* = 65.33, 62.5% female, 37.5% male). After accounting for participants who did not have memory data due to dropping out of the study (14 YA, 9 OA) or not completing the emotional memory task (2 YA), 168 participants were included in our analyses. Sample size was determined to sufficiently power the clinical trial main outcomes, which were functional magnetic resonance imaging (MRI) measures of emotion brain networks. We planned to enroll 208 participants but had to terminate the trial before older adult data collection was complete, due to COVID. A total sample size of 128 provides 80% power to detect medium effect sizes (*d* = 0.5) at *α* = 0.05, for two tailed *t*-tests, as well as for 2 (age) × 2 (condition) *F* test interactions (Faul et al., 2009). Thus, with *N* = 168, we have more than sufficient power to detect medium effect sizes for the current study. See Supplementary Fig. 1 for a detailed breakdown by condition and any inclusions and exclusions. Age, education, and gender were similar in the two conditions (Tables 1 and 2).

**Table 1:** Age, sex, and education by condition and age group

	Age		Years of Education		Sex	
	Mean (SD)	Min–Max	Mean (SD)	Min–Max	Female	Male
OSC+ YA (*N* = 55)	22.78 (2.44)	18–28	16.06 (1.76)	12–20	*N* = 26	*N* = 29
OSC− YA (*N* = 50)	22.68 (3.13)	18–31	15.82 (2.59)	12–24	*N* = 25	*N* = 25
OSC+ OA (*N* = 32)	64.88 (8.08)	55–80	16.72 (2.45)	13–25	*N* = 22	*N* = 10
OSC− OA (*N* = 31)	64.90 (5.58)	55–77	16.34 (2.25)	12–22	*N* = 21	*N* = 10

*OSC+* HRV increase condition, *OSC−* HRV decrease condition, *YA* younger adults, *OA* older adults

**Table 2:** Race of participants by condition and age group

	OSC+ YA (*N* = 55)	OSC− YA (*N* = 50)	OSC+ OA (*N* = 32)	OSC− OA (*N* = 31)
African American	4		9	2
Asian	40	34	5	6
Caucasian	7	12	16	21
More than one race	1	1	2	1
Other	2	3		
Prefer not to state	1			1

*OSC+* HRV increase condition, *OSC−* HRV decrease condition, *YA* younger adults, *OA* older adults

Participants were recruited via the USC Healthy Minds community subject pool, a USC online bulletin board, Facebook, flyers, targeted mailing campaigns, and online advertisements on volunteer websites. Prospective participants were screened to identify healthy adults (1) who did not have a major medical, neurological, or psychiatric illness, (2) who were not practicing meditation or any breathing techniques, and (3) who were not taking any psychoactive drugs with the exception of antidepressants or anti-anxiety medications (treatment must have been ongoing for a minimum of 3 months and not expected to change). Individuals who would have difficulty completing the biofeedback practice (e.g., due to coronary artery disease, angina, cardiac pacemaker, etc.) or who would be unable to complete MRI scans were excluded.

Participants were assigned to small groups of 3–7 individuals, and after groups were scheduled and confirmed, they were randomized to either the Osc+ or Osc− condition by flipping a coin. Each group met in the lab once a week on the same day at the same time over the course of 7 weeks. Participants were compensated upon completion of the study, along with additional rewards based on their individual and/or group performances. For each lab visit, participants received $15 per hour and could earn additional rewards based on individual (up to $20 per week) and group performance ($6–$18 per week depending on group size). Researchers calculated rewards weekly and provided individual updated earnings totals at each lab visit.

### Study Protocol Overview and Emotional Memory Task

As mentioned previously, we were interested in how biofeedback training affected positive emotional memory bias and how this bias related to the changes in resting amygdala-mPFC functional connectivity. Data for the emotional memory task was collected as part of a larger 7-week intervention study, in which participants completed 5 weeks of biofeedback training (more information on biofeedback training described below). Participants completed questionnaires assessing mood and anxiety at every lab visit and completed various cognitive tasks and an MRI scan at Week 2 (pre-intervention) and Week 7 (post-intervention) of the study. We discuss the full study protocol, emotion and MRI outcomes in a separate paper (Nashiro et al., 2022). The current paper examines the effects of the HRV biofeedback training intervention on emotional memory. Daily biofeedback training (20–40 min per day) started in Week 2, and researchers held group check-ins with participants in subsequent weeks to discuss progress with and questions about the training. In addition, a customized app provided daily practice reminders, positive feedback and allowed participants to encourage other members of their group from home. The emotional memory task was administered over two lab visits, during Week 4 and Week 5. Since participants began their daily biofeedback training starting on different days in Week 2, they completed 2.5–3 weeks of training prior to the recall and recognition task administered in Week 5.

The emotional memory task utilized stimuli from The Nencki Affective Picture System (NAPS) (Marchewka et al., 2014), which is a database of realistic photographs that aim to induce positive, negative, or neutral emotional states. Seventy-two stimuli were selected, counterbalanced by valence (24 each of positive, negative, and neutral). Two sets of 36 stimuli were then created, again counterbalanced by valence in each set (12 each of positive, negative, and neutral). Each positive, negative and neutral subset in the first set did not significantly differ in its mean valence and arousal from the corresponding subset in the second set. The Qualtrics Survey platform was used to administer the memory task.^1^

As mentioned above, the emotional memory task was administered over two lab visits, during Week 4 and Week 5. At the Week 4 visit, participants viewed, in random order, one of the two sets of emotional stimuli with the set selected randomly for each participant. After viewing each image, participants were asked to rate how positive, negative, or neutral they found the images on a 1–9 scale (1 = *very negative* to 9 = *very positive*, with 5 = *neutral*). Each image was displayed on the screen for 3 s, and there was no time limit for providing their ratings. After the participant viewed and rated all images in the set, they were given a free recall task in which they were asked to describe in detail as many images as they could that they just viewed. There was no time limit imposed for the free recall.

When participants returned for their Week 5 visit, they were first given the same free recall task and same instructions as in Week 4. After completing the free recall, participants completed a recognition test, where they viewed, in random order, the 36 stimuli seen during Week 4 intermixed with 36 previously unseen stimuli. As part of this recognition test, we asked participants to rate the subjective vividness of their memories using the Remember/Know (RK) paradigm (Tulving, 1985). This rating scheme assesses the difference between a rich recollection and familiarity; when presented with a stimulus, participants respond with Remember when the memory is vividly recollected and with Know when the memory is confidently recognized but without any particular or vivid detail.

Thus, for the recognition test, participants were given 3 response options for each image: Remember, Know, and New. “Remember” was described as having a vivid memory of an image, such that it could evoke thoughts or feelings when it was seen, recollections of something else that happened at that same moment, or where in sequence the image was. “Know” was described as being confident in having seen the image but that nothing specific related to thoughts, feelings, or experience could be associated with the image. “New” was described as being confident that the image was not seen before. Each image was displayed on the screen for 3 s, and there was no time limit for providing their responses.

### HRV Biofeedback Calibration and Training

All participants received an ear sensor to measure their pulse and a small laptop with software to complete biofeedback sessions at the Week 2 lab visit. At lab visits, participants’ resting HRV (summarized as RMSSD [root mean square of successive differences]) was first measured which was followed by a short calibration session (for the full calibration description, see Nashiro et al., 2022). Briefly, the purpose of the calibration session was to determine the best resonance frequency that maximizes heart rate oscillations for Osc+ participants (note that resonance frequency is different for each person and may slightly fluctuate between weeks) or to determine the best strategy to decrease heart oscillations for Osc− participants. After each calibration session at lab visits, participants completed daily biofeedback training at home using the best resonance frequency or strategy determined in their most recent lab visit (for the full home training description, see Nashiro et al., 2022).

For Osc+ participants, biofeedback training was facilitated by the emWave Pro software (Heartmath, 2016), which provided real-time heart rate biofeedback as participants inhaled through the nose and exhaled through the mouth while following a visual pacer. At the end of each home biofeedback training session, the software provided a summary “coherence” score. Heart rate oscillatory activity at a slow breathing frequency was reflected in high coherence scores; thus, participants were instructed to aim for high coherence (for more details see Nashiro et al., 2022).

For Osc− participants, biofeedback training was facilitated by a custom-developed software (Feng, 2018), which provided positive feedback for low coherence scores as participants employed various strategies to lower their heart rate oscillatory activity. Examples of strategies included imagining a scene or an out-of-body experience. At the end of each home biofeedback training session, the software provided a “calmness” score (an “anti-coherence” score). Higher calmness scores reflected lower heart rate oscillatory activity; thus, participants were instructed to aim for a high calmness score (for more details see Nashiro et al., 2022).

In general, the short calibration breathing sessions took place before the emotional memory task was administered, except for situations where there were scheduling challenges with the participant. After completing the emotional memory task, participants completed one 20-min biofeedback session at their resonance frequency. For Osc+ participants, 53% started this biofeedback session within 15 min of ending the emotional memory task. During the Week 4 session, none of the Osc+ participants completed the biofeedback before the emotional memory task, while one participant completed the memory task first before calibration. During the Week 5 session, three Osc+ participants completed the biofeedback before the memory task, while four participants completed the memory task first before calibration. For Osc− participants, 59% started the 20-min biofeedback session within 15 min of ending the emotional memory task. Due to errors in capturing timestamps for tasks, we were unable to calculate the number of minutes between the end of the memory task and start of the biofeedback session for 1% of participants. During the Week 4 session, three Osc− participants completed the biofeedback before the emotional memory task, while two participants completed the memory task first before calibration. During the Week 5 session, four Osc− participants completed the biofeedback before the memory task, while five participants completed the memory task first before calibration.

### Resting Amygdala-mPFC Functional Connectivity

We employed a 3 T Siemens MAGNETOM Trio scanner with a 32-channel head array coil at the USC Dana and David Dornsife Neuroimaging Center. T1-weighted 3D structural MRI brain scans were acquired using a magnetization prepared rapid acquisition gradient echo (MPRAGE) sequence with *TR* = 2300 ms, *TE* = 2.26 ms, slice thickness = 1.0 mm, flip angle = 9°, field of view = 256 mm, and voxel size = 1.0 × 1.0 × 1.0 mm, with 175 volumes collected (4:44 min). Resting state functional MRI was acquired using multi-echo-planar imaging sequence with *TR* = 2400 mm, TE 18/35/53 ms, slice thickness = 3.0 mm, flip angle = 75°, field of view = 240 mm, voxel size = 3.0 × 3.0 × 3.0 mm, 175 volumes and acquisition time = 7 min. Participants were instructed to rest, breathe normally and look at the central white cross on the black screen. We used multi-echo sequences to minimize the effects of motion and physiological effects. The multi-echo sequences helped remove non-BOLD signal, including signal from the basal vein of Rosenthal that often is a confounding factor in amygdala connectivity analyses (for details, see Nashiro et al., 2022, Supplementary Fig. 4).

The mPFC was defined based on a previous meta-analysis of brain regions where activity correlated with HRV (a sphere of 10 mm around the peak voxel, *x* = 2, *y* = 46, *z* = 6; Thayer et al., 2012). The left amygdala was anatomically defined using that participant’s T1 image. The segmentation of the left amygdala was performed using the FreeSurfer software package version 6 using the longitudinal processing scheme implemented to incorporate the subject‐wise correlation of longitudinal data into the processing stream (for more details, see Nashiro et al., 2022). We applied a low-pass temporal filter 0–0.1 Hz and extracted time series from the mPFC. For each participant, a multiple regression analysis was performed in FSL FEAT with nine regressors including the mPFC time series, signal from white matter, signal from cerebrospinal and six motion parameters. The individual amygdalae were registered to the standard MNI 2-mm brain using FSL FLIRT using trilinear interpolation followed by a threshold of 0.5 and binarize operation with fslmaths to keep the mask a similar size. From each participant’s mPFC connectivity map, we extracted the mean beta values from the left amygdala, which represents the strength of functional connectivity with mPFC.

## Results

### Recall Coding Procedures and Inter-rater Reliability

Two researchers reviewed and coded each participant’s recall data to ensure inter-rater reliability. Each researcher separately reviewed the description of images that participants recalled. If the description matched one from the set of images that the participants viewed the previous week, the researcher noted the name of the image. If it did not match, the researcher noted this as well. Once both researchers finished coding the recall data in this way, a third researcher compiled both coded responses, checked for discrepancies, and noted these instances. The two coders together then reviewed the discrepancies and resolved them by coming to an agreement for the final coding. Inter-rater reliability was computed using Krippendorff’s alpha (Hayes & Krippendorff, 2007). Each picture in the stimuli set was coded for each participant as reported (1, occasionally 2 if two recalled descriptions were coded as matching the same picture or 0). Krippendorff’s alpha was excellent for both Week 4 (*M* = 0.907, 95% CI [0.889, 0.927]) and Week 5 recall (*M* = 0.889, 95% CI [0.858, 0.920]).

### Data Exclusions

Four participants were excluded from recall analyses due to errors in task completion. Thus, 164 participants remained for analyses of recall (YA Osc+ *N* = 54, YA Osc− *N* = 50; OA Osc+ *N* = 29, OA Osc− *N* = 31).^2^ Three participants were excluded from recognition analyses due to errors in task completion. Thus, 165 participants remained for analyses of recognition performance (YA Osc+ *N* = 55, YA Osc− *N* = 50; OA Osc+ *N* = 29, OA Osc− *N* = 31). Twenty-one participants were excluded from mediation analyses because they did not complete a post-intervention MRI scan, and the same four participants who were excluded from recall analyses were also excluded from mediation analyses. Thus, 143 participants remained for mediation analyses (YA Osc+ *N* = 49, YA Osc− *N* = 46; OA Osc+ *N* = 23, OA Osc− *N* = 25).

### Week 4 Image Ratings

A 2 (Condition: Osc+ vs. Osc−) × 3 (Average Valence Ratings: Neutral vs. Positive vs. Negative) mixed-design factorial analysis of variance (ANOVA) resulted in no significant interaction between condition and valence, *F*(2, 328) = 0.373, *p* = 0.689). There were no differences between conditions in the average Week 4 ratings for neutral, positive and negative images, *t*(164) = 0.177, *p* = 0.860, *t*(164) = − 0.148, *p* = 0.883, and *t*(164) = 0.947, *p* = 0.345, respectively.

### Emotional Bias Score

To examine if the intervention-induced left amygdala-mPFC functional connectivity changes mediated any changes in positive emotional bias at the time of memory retrieval, we created a summary emotional memory bias score with Week 5 false recognition and Week 5 recall contributing equally to the formula. In recognition, differential false alarm rates across different categories of new items (e.g., positive, neutral, and negative) indicate a bias favoring one category over another, whereas differential hit rates to previously seen images from different categories can reflect either differences in accurate memory or differences in the bias to respond ‘old’ (i.e., that they have seen an image before). Thus, we focused on the false alarm rates as the best available measure of recognition bias. As the recognition test was only administered on Week 5, to be parallel with the recognition measure, we used the Week 5 recall and not the Week 4 recall in this summary score. While differential recall across picture valence categories could reflect valence-specific effects at any stage in the encoding, consolidation or retrieval process, by focusing on Week 5 when veridical memory would be weaker than in Week 4, we should be giving the most scope for retrieval biases to operate (Pezdek & Roe, 1995; Stahlberg & Maass, 1997).

First, the *Z* scores for positive and negative false alarms and correct recall counts were computed across participants. That is, the average false alarm rates and correct recall counts for positive and negative images were set to zero and variability was normalized such that scores of ± 1 reflected a bias one standard deviation from the mean for that type of item. The positive emotional memory bias score was then computed with the following formula: (Z FalseAlarmPositive—Z FalseAlarmNegative) + (Z CorrectRecallPositive—Z CorrectRecallNegative).

We used this positive emotional bias score as the dependent measure in a 2 (Condition: Osc+ vs. Osc−) × 2 (Age: YA vs. OA) univariate ANOVA. There was a significant effect of condition, *F*(1,160) = 5.37, *p* = 0.022, *η*_*p*_^*2*^ = 0.032, and no other significant effects. The Osc+ condition showed more positive bias (*M* = 0.209, 95% CI [− 0.087, 0.506]) than the Osc− condition (*M* = − 0.281, 95% CI [− 0.575, 0.014]).

### Mediating Role of Intervention-Induced Changes in Left Amygdala-mPFC Functional Connectivity on Effects of Condition on Positive Emotional Memory Bias

To examine if the intervention-induced left amygdala-mPFC functional connectivity changes mediated any changes in positive emotional memory bias, we conducted a mediation analysis using Model 4 of the PROCESS macro (Hayes, 2017). The mediation analysis was conducted using bootstrapping and a resampling procedure of 10,000 bootstrap samples (Preacher & Hayes, 2008). Point estimates (effect size estimates) and confidence intervals (95%) were estimated for the indirect effect. If the confidence interval did not contain zero, the point estimate was considered significant, as zero indicates no indirect effects of the independent variable (biofeedback condition) on the dependent variable (emotional memory bias) through the proposed mediator (mPFC-left amygdala functional connectivity).

**Fig. 2:**
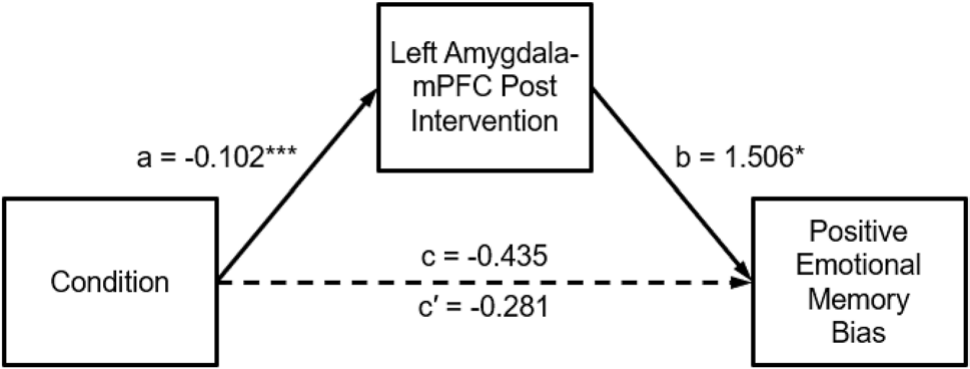
Left amygdala-mPFC mediation model of the relationship between condition and positive emotional memory bias. *p < .05, ***p < .001

As shown in the mediation analysis diagram (Fig. 2), the unstandardized regression coefficient c represents total effect, coefficient c’ represents the direct effect of the independent variable on the dependent variable without any mediator, coefficient a represents the relationship between mediator and the independent variable, and coefficient b represents the relationship between the mediator and the dependent variable. The product of coefficients a and b (a × b) represents the indirect effect, reflecting the degree to which the mediator accounts for the relationship between the independent and dependent variable. The direct, indirect, and total effects of the model including 95% confidence intervals from the bootstrapping method are reported in Table 3.

**Table 3:** Path coefficients for mediation model (*N* = 143, bootstrap = 10,000)

Effect	Paths	B	SE	t	p	LLCI	ULCI
Total effect (c)	Condition → positive emotional memory bias	− 0.435	0.225	− 1.930	.056	− 0.880	0.011
Direct effect (c′)	Condition → positive emotional memory bias	− 0.281	0.230	− 1.222	.224	− 0.737	0.174
Indirect effect (ab)	Condition → left-amygdala-mPFC post-intervention → positive emotional memory bias	− 0.153	0.066			− 0.289	− 0.031

We examined the mediation model depicted in Fig. 2, controlling for left amygdala-mPFC connectivity at pre-intervention. With positive emotional memory bias as the dependent variable (Fig. 2 and Table 3), the total effect was not statistically significant, *c* = − 0.435, *p* = 0.056, 95% CI [− 0.880, 0.011]. The direct effect was not significant, c′ = − 0.281, *p* = 0.224, 95% CI [− 0.737, 0.174], but the indirect effect was significant, ab = − 0.153, 95% CI [− 0.289, − 0.031]. Thus, the mediation model indicates that changes in left amygdala-mPFC functional connectivity significantly mediated the effects of condition on positive emotional memory biases.^3^

While these findings suggest that the interventions affected positive emotional memory biases by influencing brain circuits involved in emotion regulation, it is also possible that both the left amygdala-mPFC functional connectivity and the emotional memory biases depended on some other intervention-related change, such as in mood or resting HRV. Indeed, higher baseline RMSSD was associated with higher positive emotional memory bias, *r*(160) = 0.198, *p* = 0.012, but baseline mood was not significantly associated with positive emotional memory bias, *r*(164) = − 0.005, *p* = 0.952.^4^ We conducted follow-up analyses to check whether the left amygdala-mPFC functional connectivity changes still mediated condition-related effects on positive emotional memory biases when mood and resting HRV were controlled for.

First, we added change in mood as a covariate to the mediation model in Fig. 2. The Profile of Mood States (POMS) was administered at all lab visits to assess mood, and a change in mood score was computed: average of Week 4 and 5 scores—average of Week 1 and 2 scores. After accounting for changes in mood as a covariate, the total effect was not statistically significant, *c* = − 0.426, *p* = 0.062, 95% CI [− 0.873, 0.021]. The direct effect was not significant, c′ = − 0.256, *p* = 0.270, 95% CI [− 0.713, 0.201], but the indirect effect was significant, ab = − 0.170, 95% CI [− 0.320, − 0.039]. The significant indirect effect suggests that the mPFC-left amygdala functional connectivity changes mediated the condition-related effects on positive emotional memory biases, after controlling for mood. We then added change in resting HRV (RMSSD) as a covariate to the original mediation model. Resting HRV was assessed at all lab visits starting in Week 2, and a change in resting HRV score was computed: average of Week 4 and 5 RMSSD—Week 2 RMSSD. With resting HRV as a covariate, the total effect was not statistically significant, *c* = − 0.357, *p* = 0.129, 95% CI [− 0.818, 0.105]. The direct effect was not significant, c′ = − 0.214, *p* = 0.370, 95% CI [− 0.683, 0.256], but the indirect effect was significant, ab = − 0.143, 95% CI [− 0.286, − 0.021]. Similar to the results when controlling for mood, the significant indirect effect suggests that the mPFC-left amygdala functional connectivity changes mediated the condition-related effects on positive emotional memory biases, after controlling for resting HRV.

The results of the mediation analysis suggest that the relationship between condition and positive emotional memory bias is fully mediated by the left amygdala-mPFC functional connectivity at post-intervention, due to the statistically significant indirect effect, even after controlling for changes in mood and resting HRV. As such, the increase in left amygdala-mPFC functional connectivity at post-intervention appeared to play a role in the intervention condition’s effects on positive emotional memory bias.

### Recognition Results

In this and the next section, we report on the complete recognition and recall findings from the study. A 2 (Condition: Osc+ vs. Osc−) × 2 (Age: YA vs. OA) × 3 (Valence: Neutral vs. Positive vs. Negative) mixed-design factorial analysis of variance (ANOVA) was performed on recognition memory measures. While our main aim is to see if there were any condition-by-valence interactions, we will first report other significant effects. There were significant main effects of valence and age on hits (proportion of old items called old), false alarms (proportion of new items called old), and corrected recognition (hits minus false alarms), as reported in Tables 4 and 5, respectively. There were no significant interactions with age for hits, false alarms, or corrected recognition. While there were no significant interactions with condition for hits or corrected recognition, there was a significant interaction with false alarms.^5^

**Table 4:** Main effects of valence for the recognition task

Recognition result	Main effects	*P* value	Neutral mean [95% CI]	Positive mean [95% CI]	Negative mean [95% CI]
False alarms	Valence	.006	0.057 [0.047, 0.067]	0.061 [0.051, 0.072]	0.047 [0.039, 0.056]
Hits	Valence	< .001	0.860 [0.844, 0.875]	0.856 [0.841, 0.870]	0.890 [0.876, 0.903]
Corrected recognition	Valence	< .001	0.803 [0.780, 0.825]	0.794 [0.772, 0.817]	0.842 [0.823, 0.861]

The 2 (Condition: Osc+ vs. Osc−) × 2 (Age: YA vs. OA) × 3 (Valence: neutral vs. positive vs. negative) ANOVA showed main effects of valence with false alarms (proportion of new items called old), hits (proportion of old items called old), and corrected recognition (hits minus false alarms). Numbers in brackets reflect 95% confidence intervals

**Table 5:** Main effects of age for the recognition task

Recognition result	Main effects	*P* value	Younger adult mean [95% CI]	Older adult mean [95% CI]
False alarms	Age	< .001	0.041 [0.032, 0.051]	0.069 [0.056, 0.082]
Hits	Age	.004	0.886 [0.872, 0.901]	0.850 [0.831, 0.870]
Corrected recognition	Age	< .001	0.845 [0.824, 0.867]	0.781 [0.753, 0.810]

The 2 (Condition: Osc+ vs. Osc−) × 2 (Age: YA vs. OA) × 3 (Valence: neutral vs. positive vs. negative) ANOVA showed main effects of age with false alarms, hits, and corrected recognition. Numbers in brackets reflect 95% confidence intervals

**Fig. 3:**
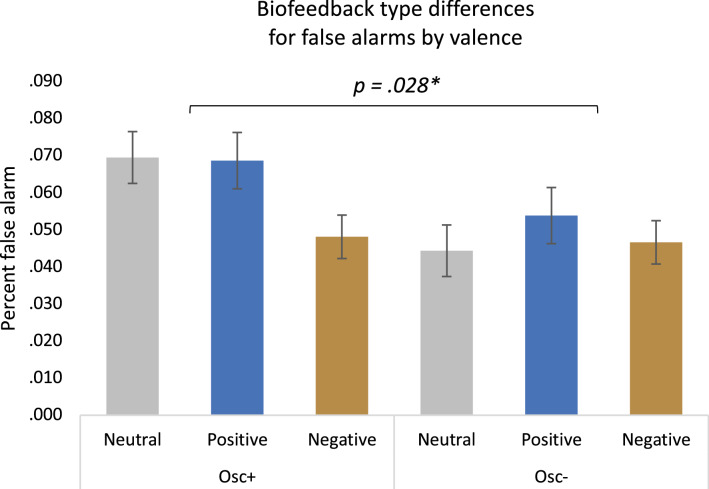
False alarms for all participants during the recognition memory task. There was a significant interaction of condition and valence. Osc+ participants had a higher rate of false alarm for neutral and positive images than negative images, while Osc− participants showed no differences in false alarm rate for neutral, positive, or negative images

Overall, there was a significant main effect of valence on false alarms, *F*(2,322) = 5.21, *p* = 0.006, *η*_*p*_^*2*^ = 0.031, see Table 4 for means and CIs. False alarms were higher for positive images compared with negative images and were also higher for neutral images than negative images. There was also a significant interaction between condition and valence, *F(*2,322) = 3.63, *p* = 0.028, *η*_*p*_^*2*^ = 0.022 (Fig. 3 and Table 6). In the Osc+ condition, there was a higher rate of false alarm responses for neutral (*M* = 0.070, 95% CI [0.056, 0.083]) than negative images (*M* = 0.048, 95% CI [0.037, 0.060]). There was also a higher rate of false alarms for positive images (*M* = 0.069, 95% CI [0.054, 0.084]) than negative images (*M* = 0.048, 95% CI [0.037, 0.060]). Conversely, for Osc− participants, there was no significant difference in false alarm rates for neutral (*M* = 0.044, 95% CI [0.031, 0.058]), positive (*M* = 0.054, 95% CI [0.039, 0.069]), nor negative images (*M* = 0.047, 95% CI [0.035, 0.058]). Additionally, there was a significant interaction between condition and age category, *F(*1,161) = 4.12, *p* = 0.044, *η*_*p*_^*2*^ = 0.025, for false alarms. Older adults in the Osc+ condition (*M* = 0.084, 95% CI [0.066, 0.103]) had a higher false alarm rate than older adults in the Osc− condition (*M* = 0.054, 95% CI [0.036, 0.072]). However, this difference was not observed among the younger adults when comparing the Osc+ condition (*M* = 0.040, 95% CI [0.026, 0.053]) and the Osc− condition (*M* = 0.043, 95% CI [0.029, 0.057]).

**Table 6:** Mean false alarm responses by valence, age, and condition

Condition	False alarm responses		
Condition	Neutral mean [95% CI]	Positive mean [95% CI]	Negative mean [95% CI]
OSC+ (YA + OA)	.070 [.056, .083]	.069 [.054, .084]	.048 [.037, .060]
OSC+ (YA)	.048 [.032, .065]	.044 [.026, .062]	.027 [.014, .041]
OSC+ (OA)	.091 [.068, .113]	.093 [.069, .118]	.069 [.050, 0.88]
OSC− (YA + OA)	.044 [.031, .058]	.054 [.039, .069]	.047 [.035, .058]
OSC− (YA)	.042 [.025, .059]	.046 [.027, .064]	.041 [.027, .055]
OSC− (OA)	.047 [.025, .069]	.062 [.038, .085]	.052 [.034, .071]

Numbers in brackets reflect 95% confidence intervals

*OSC+* HRV increase condition, *OSC−* HRV decrease condition, *YA* younger adults, *OA* older adults

Follow-up ANOVAs were performed to determine if responses in the two biofeedback conditions differed by Remember or Know for false alarms, hits, or corrected recognition. We used a 2 (Condition: Osc+ vs. Osc−) × 2 (Age: YA vs. OA) × 3 (Valence: neutral vs. positive vs. negative) × 2 (Response: Remember vs. Know) ANOVAs performed for each memory measure. These analyses did not reveal any condition differences in the relative Remember vs. Know judgment rates. Overall, there were significant main effects of response for false alarms, hits, and corrected recognition. For false alarms, Know responses were higher (*M* = 0.083, 95% CI [0.069, 0.096]) than Remember ones (*M* = 0.028, 95% CI [0.020, 0.036]), *F*(1,161) = 47.24, *p* < 0.001, *η*_*p*_^*2*^ = 0.227. For hits and corrected recognition, the opposite results were found. For hits, Remember responses were higher (*M* = 0.557, 95% CI [0.521, 0.592]) than Know ones (*M* = 0.291, 95% CI [0.261, 0.320]), *F*(1,161) = 71.24, *p* < 0.001, *η*_*p*_^*2*^ = 0.307. For corrected recognition, Remember responses were higher (*M* = 0.529, 95% CI [0.494, 0.564]) than Know ones (*M* = 0.208, 95% CI [0.175, 0.241]), *F*(1,161) = 98.92, *p* < 0.001, *η*_*p*_^*2*^ = 0.381.

As expected based on the aforementioned 2 (Condition: Osc+ vs. Osc−) × 2 (Age: YA vs. OA) × 3 (Valence: neutral vs. positive vs. negative) ANOVA results reported above, there was a significant main effect of valence for false alarms responses, *F*(2,322) = 5.21, *p* = 0.006, *η*_*p*_^*2*^ = 0.031, a significant interaction between condition and valence, *F*(2,322) = 3.63, *p* = 0.028, *η*_*p*_^*2*^ = 0.022, as well as a significant interaction between condition and age category, *F*(1,161) = 4.12, *p* = 0.044, *η*_*p*_^*2*^ = 0.025. To review the false alarm means, please refer above to the parallel analyses without Remember vs. Know response as a factor; the *F* values and cell means are identical for these effects not involving response as a factor. There were no other significant interactions with the intervention condition. Likewise, there were no significant interactions between condition and response type (Remember vs. Know) for hits or corrected recognition. Thus, in summary, the analyses including Remember vs. Know as a factor are consistent with the other analyses and do not indicate any effects of condition on the subjective vividness of memories.

## Recall Results

For recall, a 2 (Condition: Osc+ vs. Osc−) × 2 (Age: YA vs. OA) × 3 (Valence: Neutral vs. Positive vs. Negative) × 2 (Time: Week 4 vs. Week 5) mixed-design factorial analysis of variance (ANOVA) with recall counts as the dependent measures was performed. There was a main effect of time, *F*(1, 160) = 456.029, *p* < 0.001, *η*_*p*_^2^ = 0.740, as the number of recalled images that was emotional was overall higher at Week 4 (*M* = 4.016, 95% CI [3.714, 4.317]) than Week 5 (*M* = 1.783, 95% CI [1.556, 2.010]). There was also a main effect of valence, *F*(2, 320) = 6.92, *p* = 0.001, *η*_*p*_^2^ = 0.041, as participants recalled a higher number of positive (*M* = 2.916, 95% CI [2.638, 3.193]) and negative (*M* = 3.141, 95% CI [2.848, 3.433]) images compared to neutral images (*M* = 2.642, 95% CI [2.343, 2.941]). The average number of correctly recalled images (by valence) and incorrectly recalled images for both Osc+ and Osc− conditions are presented in Table 7.

**Table 7:** Average number of correctly and incorrectly recalled images during Week 4 and 5 by condition and age category

Condition and age category	Correctly recalled images			Incorrectly recalled images
	Neutral mean [95% CI]	Positive mean [95% CI]	Negative mean [95% CI]	All valences [95% CI]
Week 4 recall				
OSC+ YA (*N* = 55)	3.582 [2.962, 4.201]	4.618 [4.022, 5.214]	4.673 [4.060, 5.285]	1.436 [0.962, 1.911]
OSC− YA (*N* = 50)	3.260 [2.610, 3.910]	4.200 [3.575, 4.825]	4.220 [3.578, 4.862]	1.460 [0.963, 1.957]
OSC+ OA (*N* = 30)	3.600 [2.761, 4.439]	3.633 [2.826, 4.440]	3.700 [2.871, 4.529]	2.633 [1.991, 3.275]
OSC− OA (*N* = 31)	3.968 [3.142, 4.793]	4.258 [3.464, 5.052]	4.871 [4.055, 5.686]	2.677 [2.046, 3.309]
Week 5 recall				
OSC+ YA (*N* = 54)	2.093 [1.635, 2.550]	2.426 [1.982, 2.870]	2.204 [1.731, 2.667]	1.741 [1.209, 2.272]
OSC− YA (*N* = 50)	1.700 [1.225, 2.175]	1.740 [1.279, 2.201]	1.980 [1.488, 2.472]	1.880 [1.327, 2.433]
OSC+ OA (*N* = 29)	1.103 [0.479, 1.727]	1.379 [0.773, 1.985]	1.517 [0.872, 2.163]	2.483 [1.757, 3.208]
OSC− OA (*N* = 31)	1.871 [1.267, 2.474]	1.355 [0.769, 1.941]	2.032 [1.408, 2.657]	2.613 [1.911, 3.315]

One hundred sixty-six participants recalled images at the Week 4 visit while 164 participants recalled images at Week 5. Correctly recalled images were identified by coders and researchers matched them against the NAPS image and valence. Incorrectly recalled images were not matched against the NAPS image

Numbers in brackets reflect 95% confidence intervals

There were no significant within-subjects interactions by condition. However, there was a significant interaction between time and valence, *F*(2, 320) = 6.15, *p* = 0.002, *η*_*p*_^*2*^ = 0.037. Examination of the means and confidence intervals indicate that the number of recalled images declined more from Week 4 (*M* = 4.106, 95% CI [3.759, 4.454]) to Week 5 (*M* = 1.725, 95% CI [1.460, 1.990]) for positive images and from Week 4 (*M* = 4.348, 95% CI [3.980, 4.716]) to Week 5 (*M* = 1.933, 95% CI [1.651, 2.215]) for negative images compared to neutral images from Week 4 (*M* = 3.592, 95% CI [3.218, 3.967]) to Week 5 (*M* = 1.692, 95% CI [1.419, 1.964]).

Finally, there was a significant between-subjects interaction between condition and age category, *F*(1,160) = 3.942, *p* = 0.049, *η*_*p*_^*2*^ = 0.024. Younger adults in the Osc+ condition (*M* = 3.235, 95% CI [2.822, 3.647]) recalled more images than younger adults in the Osc− condition (*M* = 2.850, 95% CI [2.421, 3.279]). Conversely, older adults in the Osc+ condition (*M* = 2.454, 95% CI [1.891, 3.017]) recalled fewer images than older adults in the Osc− condition (*M* = 3.059, 95% CI [2.514, 3.604]).

## Discussion

The findings reported in this paper suggest that performing daily biofeedback practice to increase heart oscillations impacts positive emotional bias during memory retrieval by way of implicit emotion regulation in the left amygdala-mPFC. In this clinical trial, participants who were instructed to increase heart rate oscillatory activity through daily biofeedback training (Osc+ condition) had a memory bias favoring positive over negative images compared with participants who were instructed to use daily biofeedback to reduce heart rate oscillatory activity (Osc− condition).

We previously found that the Osc+ condition increased left amygdala-mPFC resting-state functional connectivity, whereas the Osc− condition did not affect this emotion-regulation-related relationship (Nashiro et al., 2022). Here, we report new findings that increased left amygdala-mPFC functional connectivity at post-intervention mediated the relationship between Osc ± condition and a summary score representing positive > negative emotional memory bias based on both recognition false alarms and recall. The mediation analysis (which controlled for baseline left amygdala-mPFC functional connectivity) did not result in significant total effects or direct effects, although there was a trend towards a total effect. However, there was a significant indirect effect. Thus, intervention-induced changes in mPFC-left amygdala functional connectivity were associated with positive emotional memory bias.

In addition to computing a summary positive emotional memory bias score to be able to complete the mediation analysis, we also reported the separate recognition and recall results. The Osc+ condition had a higher rate of false alarms for positive and neutral images than negative images, whereas the Osc− condition did not show any differences with regard to image valence. No condition interactions were seen for hits or corrected recognition or for relative proportions of Remember vs. Know responses. That the effects were seen for false alarms but not for hits suggests that at least part of the effect of HRV-biofeedback on positive emotional memory is due to decision criteria operating during retrieval. There were no significant condition-by-valence interactions on the number of items recalled, although numerically the Osc+ participants showed more of a positive > negative bias than the Osc− participants in Week 5 recall.

A strength of this study is that it included both younger and older adults. Despite differing in their baseline levels of HRV, younger and older adults showed similar effects of the HRV biofeedback interventions on emotional memory. Older adults are an interesting group to examine for this issue, as despite having lower HRV than younger adults (Umetani et al., 1998), they often show a positivity effect in memory compared with younger adults (Mather & Carstensen, 2005; Reed et al., 2014). In the current study, we failed to replicate the age-related positivity effect (i.e., an age-by-valence interaction) so did not have the opportunity to see how age differences in positivity might relate to responses to HRV biofeedback. This lack of an overall age-by-valence interaction may be because all participants had already been engaged in one of the two intervention conditions for several weeks before seeing the pictures. Also, we note that older adults in the Osc+ condition recalled fewer pictures overall and made more false alarms than those in the Osc− condition, whereas younger adults did not show this effect. This was an unexpected finding, and we are not sure why the interventions would have affected the two age groups’ overall memory accuracy differently. To shed light on these questions, in a subsequent study it would be helpful to include baseline (pre-intervention) measures of emotional memory and associated biases. It is possible that individual or group differences in overall memory or memory bias at baseline are related to the impact of HRV biofeedback.

A limitation of our study was that we did not have brain scans at each lab visit. Thus, we cannot address the question of the relative contributions of the short-term (within lab-visit session) vs. long-term (across several weeks) effects of the HRV manipulation. Although both the resting-state fMRI and the emotional memory measures were collected when participants in the two conditions were breathing normally (e.g., for breathing measures during resting fMRI see Nashiro et al., 2022) and not trying to do HRV biofeedback at that moment, they had practiced the biofeedback every day for several weeks, often within the past few hours of completing the brain scan and memory task. Thus, additional research is needed to determine the acute vs. long-term influence of HRV biofeedback on emotional memory. For instance, an interesting question for future research is whether HRV biofeedback influences amygdala-mPFC functional connectivity for the next few hours, which in turn influences memory biases during a short time frame, or whether the intervention has a longer-lasting effect that might be seen even if a participant has not practiced HRV biofeedback in the past day or so.

We found a small-to-medium effect of the intervention on positive emotional memory bias. Meta-analyses indicate that the relationship between negative self-referential implicit cognition and depression is a small-to-medium effect size (Phillips et al., 2010), the relationship between specific/overgeneral memories on the Autobiographical Memory Test and depressive symptoms at follow-up is a small effect size (Sumner et al., 2010), and greater implicit recall of negative words and reduced implicit recall of positive words among those with depression compared with non-depressed individuals were small effect sizes (Gaddy & Ingram, 2014). Thus, although not large, our effects are in the range of the memory and bias effects seen in depression.

The main strength of this study is that it goes beyond examining correlations between HRV and memory by conducting an intervention. Because participants were trained to either increase or decrease their HRV through daily practice before being assessed on a memory task, we were able to observe the potential impact that attempting to modulate HRV has on emotional memory. In addition, it was a strength of the study that we could use a functional neuroimaging outcome from the trial to probe a potential mechanism contributing to the effect. Thus, the results provide important evidence supporting theorizing that brain circuits controlling HRV are also involved in implicit emotion regulation processes (Mather & Thayer, 2018).

## Supplementary Information

Below is the link to the electronic supplementary material.

Supplementary file1 (DOCX 83 KB)

## Data Availability

All data, analysis, and research materials are available at https://osf.io/t5s4z/.

## Footnotes

1 Qualtrics was programmed to evenly, but randomly, allocate the two sets at encoding between Osc + and Osc-. Set A and B were each presented 42 times for Osc + . Set A was presented 42 times and Set B 39 times for Osc-.

2 YA = younger adults (ages 18-31), OA = older adults (ages 55-80).

3 The significance of this mediation model remains the same when age is added as a covariate.

4 The significance of these correlation results remain the same when age is added as a covariate.

5 Compared to Set B, Set A had a higher rate of hits (proportion of old items called old), F(163) = 7.517, p = .007, and corrected recognition (hits minus false alarms), F(163) = 4.748, p = .031. There was no difference for false alarm (proportion of new items called old) rate between sets, F(163) = .634, p = .427.

## References

[CR1] Bechara A (2004). Disturbances of emotion regulation after focal brain lesions. International Review of Neurobiology.

[CR2] Beer JS, Lombardo MV, Gross JJ, Gross JJ (2007). Insights into emotion regulation from neuropsychology. Handbook of emotion regulation.

[CR3] Braunstein LM, Gross JJ, Ochsner KN (2017). Explicit and implicit emotion regulation: A multi-level framework. Social Cognitive and Affective Neuroscience.

[CR4] Cho, C. (2022). *Heart rate variability and emotional memory biases*. Retrieved from https://osf.io/t5s4z

[CR5] Dalgleish T, Werner-Seidler A (2014). Disruptions in autobiographical memory processing in depression and the emergence of memory therapeutics. Trends in Cognitive Sciences.

[CR6] Everaert J, Vrijsen JN, Martin-Willett R, van de Kraats L, Joormann J (2022). A meta-analytic review of the relationship between explicit memory bias and depression: Depression features an explicit memory bias that persists beyond a depressive episode. Psychological Bulletin.

[CR7] Farahimanesh S, Moradi A, Sadeghi M (2021). Autobiographical memory bias in cancer-related post traumatic stress disorder and the effectiveness of competitive memory training. Current Psychology.

[CR8] Faul F, Erdfelder E, Buchner A, Lang AG (2009). Statistical power analyses using G*Power 3.1: Tests for correlation and regression analyses. Behavior Research Methods.

[CR9] Feng, T. (2018). Biofeedback to decrease HRV. https://github.com/EmotionCognitionLab/emWave_HRV

[CR10] Gaddy MA, Ingram RE (2014). A meta-analytic review of mood-congruent implicit memory in depressed mood. Clinical Psychology Review.

[CR11] Goessl VC, Curtiss JE, Hofmann SG (2017). The effect of heart rate variability biofeedback training on stress and anxiety: A meta-analysis. Psychological Medicine.

[CR12] Hayes AF (2017). Introduction to mediation, moderation, and conditional process analysis: A regression-based approach.

[CR13] Hayes AF, Krippendorff K (2007). Answering the call for a standard reliability measure for coding data. Communication Methods and Measures.

[CR14] Heartmath. (2016). EmWave pro plus. In https://store.heartmath.com/emwave-pro-plus/

[CR15] Kazak AE (2018). Editorial: Journal article reporting standards. American Psychologist.

[CR16] Koenig J, Abler B, Agartz I, Akerstedt T, Andreassen OA, Anthony M, Bar KJ, Bertsch K, Brown RC, Brunner R, Carnevali L, Critchley HD, Cullen KR, de Geus EJC, de la Cruz F, Dziobek I, Ferger MD, Fischer H, Flor H, Quintana DS (2021). Cortical thickness and resting-state cardiac function across the lifespan: A cross-sectional pooled mega-analysis. Psychophysiology.

[CR17] Lehrer P, Kaur K, Sharma A, Shah K, Huseby R, Bhavsar J, Sgobba P, Zhang Y (2020). Heart rate variability biofeedback improves emotional and physical health and performance: A systematic review and meta analysis. Applied Psychophysiology and Biofeedback.

[CR18] Lindquist KA, Satpute AB, Wager TD, Weber J, Barrett LF (2016). The brain basis of positive and negative affect: Evidence from a meta-analysis of the human neuroimaging literature. Cerebral Cortex.

[CR19] Marchewka A, Zurawski L, Jednorog K, Grabowska A (2014). The nencki affective picture system (NAPS): Introduction to a novel, standardized, wide-range, high-quality, realistic picture database. Behavior Research Methods.

[CR20] Mather M, Carstensen LL (2005). Aging and motivated cognition: The positivity effect in attention and memory. Trends in Cognitive Sciences.

[CR21] Mather M, Thayer J (2018). How heart rate variability affects emotion regulation brain networks. Current Opinion in Behavioral Sciences.

[CR22] Nashiro K, Min J, Yoo HJ, Cho C, Bachman SL, Dutt S, Thayer JF, Lehrer PM, Feng T, Mercer N, Nasseri P, Wang D, Chang C, Marmarelis VZ, Narayanan S, Nation DA, Mather M (2022). Increasing coordination and responsivity of emotion-related brain regions with a heart rate variability biofeedback randomized trial. Cognitive, Affective & Behavioral Neuroscience.

[CR23] Pezdek K, Roe C (1995). The effect of memory trace strength on suggestibility. Journal of Experimental Child Psychology.

[CR24] Phillips WJ, Hine DW, Thorsteinsson EB (2010). Implicit cognition and depression: A meta-analysis. Clinical Psychology Review.

[CR25] Pinna T, Edwards DJ (2020). A systematic review of associations between interoception, vagal tone, and emotional regulation: Potential applications for mental health, wellbeing, psychological flexibility, and chronic conditions. Frontiers in Psychology.

[CR26] Pizzoli SFM, Marzorati C, Gatti D, Monzani D, Mazzocco K, Pravettoni G (2021). A meta-analysis on heart rate variability biofeedback and depressive symptoms. Scientific Reports.

[CR27] Preacher KJ, Hayes AF (2008). Asymptotic and resampling strategies for assessing and comparing indirect effects in multiple mediator models. Behavior Research Methods.

[CR28] Reed AE, Chan L, Mikels JA (2014). Meta-analysis of the age-related positivity effect: Age differences in preferences for positive over negative information. Psychology and Aging.

[CR29] Sakaki M, Yoo HJ, Nga L, Lee TH, Thayer JF, Mather M (2016). Heart rate variability is associated with amygdala functional connectivity with MPFC across younger and older adults. NeuroImage.

[CR30] Samide R, Ritchey M (2021). Reframing the past: Role of memory processes in emotion regulation. Cognitive Therapy and Research.

[CR31] Stahlberg D, Maass A (1997). Hindsight bias: Impaired memory or biased reconstruction?. European Review of Social Psychology.

[CR32] Sumner JA, Griffith JW, Mineka S (2010). Overgeneral autobiographical memory as a predictor of the course of depression: A meta-analysis. Behavior Research and Therapy.

[CR33] Thayer JF, Ahs F, Fredrikson M, Sollers JJ, Wager TD (2012). A meta-analysis of heart rate variability and neuroimaging studies: Implications for heart rate variability as a marker of stress and health. Neuroscience and Biobehavioral Reviews.

[CR34] Todd RM, Cunningham WA, Anderson AK, Thompson E (2012). Affect-biased attention as emotion regulation. Trends in Cognitive Sciences.

[CR35] Tulving E (1985). Memory and consciousness. Canadian Psychology/Psychologie Canadienne.

[CR36] Umetani K, Singer DH, McCraty R, Atkinson M (1998). Twenty-four hour time domain heart rate variability and heart rate: Relations to age and gender over nine decades. Journal of the American College of Cardiology.

[CR37] Vaschillo EG, Vaschillo B, Lehrer PM (2006). Characteristics of resonance in heart rate variability stimulated by biofeedback. Applied Psychophysiology and Biofeedback.

[CR38] Yoo HJ, Thayer JF, Greening S, Lee TH, Ponzio A, Min J, Sakaki M, Nga L, Mather M, Koenig J (2018). Brain structural concomitants of resting state heart rate variability in the young and old: Evidence from two independent samples. Brain Structure & Function.

