# Supplementary material for: Changes in Medial Prefrontal Cortex Mediate Effects of Heart Rate Variability Biofeedback on Positive Emotional Memory Biases
Source: Appl Psychophysiol Biofeedback. 2023 Jan 20;48(2):135–47. doi: 10.1007/s10484-023-09579-1 (PMC10195741; doi:10.1007/s10484-023-09579-1)

**Supplementary Figure 1**

*Numbers of participants in each intervention condition, how many participants completed each measure, and how many were included vs. excluded in each analysis.*


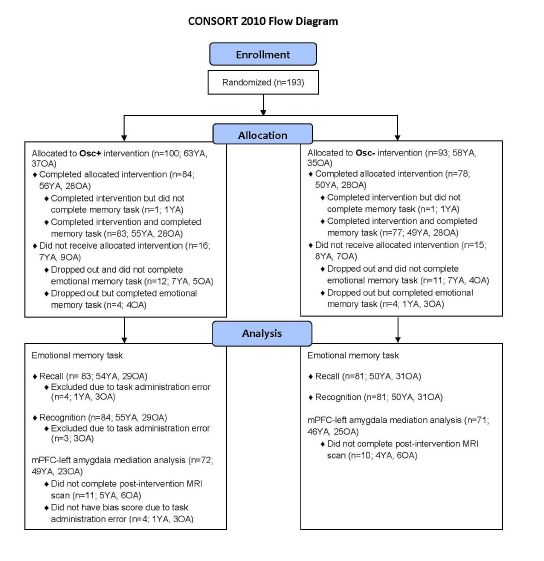

Supplement: Supplementary file 1 — Supplementary file1 (DOCX 83 KB) [file 10484_2023_9579_MOESM1_ESM.docx]
